# Laryngeal Involvement of Multiple Myeloma

**DOI:** 10.1155/2012/257814

**Published:** 2012-11-18

**Authors:** Ariel B. Grobman, Richard J. Vivero, German Campuzano-Zuluaga, Parvin Ganjei-Azar, David E. Rosow

**Affiliations:** ^1^Department of Otolaryngology, Miller School of Medicine, University of Miami, 1120 NW 14th Street, Suite 572, Miami, FL 33136, USA; ^2^Department of Pathology, Miller School of Medicine, University of Miami, 1120 NW 14th Street, 14th Floor, Miami, FL 33136, USA

## Abstract

The objectives of this paper are to discuss a rare cause of laryngeal multiple myeloma, to review unique pathologic findings associated with plasma cell neoplasms, to discuss epidemiology, differential diagnosis, and treatment options for plasma cell neoplasms of the larynx. Laryngeal multiple myeloma, also noted in the literature as “metastatic” multiple myeloma, presenting as a de novo laryngeal mass is extremely rare with few reported cases. Laryngeal involvement of extramedullary tumors is reported to be between 6% and 18% with the epiglottis, glottis, false vocal folds, aryepiglottic folds, and subglottis involved in decreasing the order of frequency. We present the case of a 58-year-old male with a history of IgA smoldering myeloma who presented to a tertiary care laryngological practice with a two-month history of dysphonia, which was found to be laryngeal involvement of multiple myeloma. We review the classification of and differentiation between different plasma cell neoplasms, disease workups, pathologic findings, and treatment options.

## 1. Introduction

A 58-year-old male presented with a two-month history of dysphonia. The physical exam demonstrated an asymmetric thyroid cartilage with a left-sided 2.5 cm firm, mobile, and nontender mass. Ten years prior, he was treated with radiotherapy for IgA myeloma involving the right acromion and lower extremity with complete resolution. Laboratory results were remarkable for serum IgA of 474 mg/dL, free kappa light chains of 7.92 mg/L, free lambda light chains of 1.96 mg/dL, a free kappa/lambda ratio of 4.04, and *β*2-microglobulin of 2.31 mg/L.

**Figure 1:**
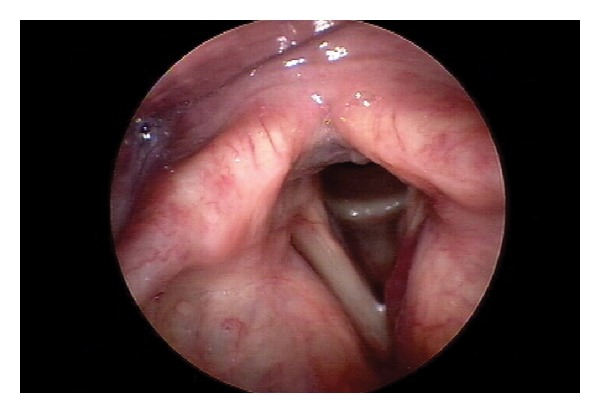
Videostroboscopic image revealing an erythematous, submucosal mass arising from and effacing the left ventricle, representing extension of the mass into the laryngeal introitus.

Videostroboscopy revealed a large submucosal mass effacing the left ventricle (Figure 1). The vocal cords appeared normal and mobile bilaterally. The position emission tomography/computerized tomography (PET/CT) revealed a hypermetabolic 3.5 × 4 cm lesion involving the left thyroid cartilage and lytic lesions in the left coracoid process and right tibia. The peripheral blood smear and bone marrow flow cytometry were unremarkable. Bone marrow failed to demonstrate abnormal populations of plasma cells or lymphocytes.

**Figure 2:**
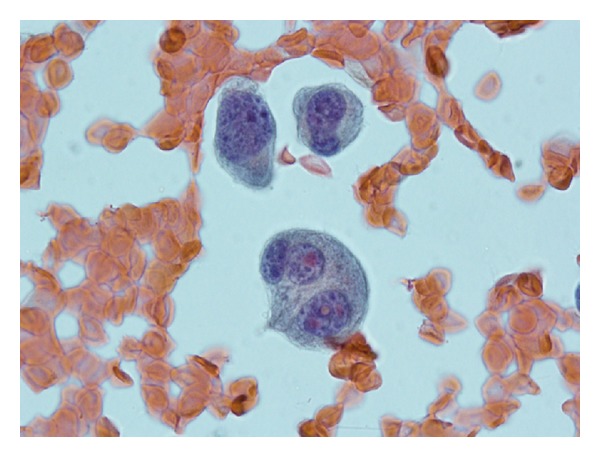
Atypical plasma cells with marked polymorphism and pleomorphism, increased nuclear-to-cytoplasmic ratio, binucleation, nuclear convolution, lobation and nuclear inclusions.

**Figure 3:**
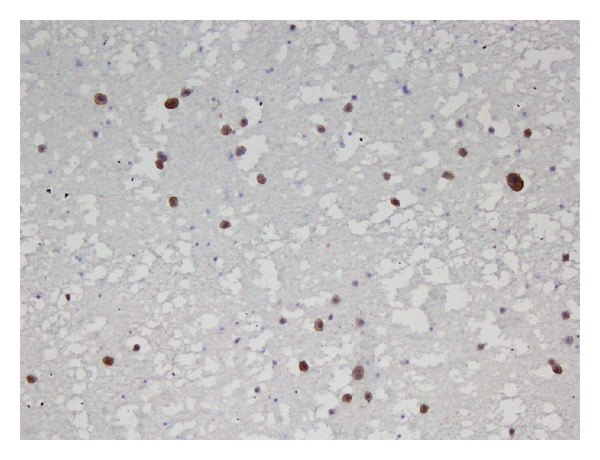
Positive CD138 immunostain obtained from fine needle aspiration of the laryngeal mass confirmed the presence of plasma cells.

A fine needle aspiration of the mass (Figure 2) showed abundant atypical plasma cells with marked pleomorphism, increased nuclear-to-cytoplasmic ratio, binucleation, nuclear convolution, lobation, and nuclear inclusions. A CD138 immunostain confirmed laryngeal involvement by a plasma cell neoplasm (Figure 3). 

## 2. Diagnosis and Discussion

Plasma cell neoplasms are clonal proliferations of immunoglobulin-producing plasma cells. Other monoclonal plasma cell neoplasms include extramedullary plasmacytoma (EMP), solitary plasmacytoma (SP), Waldenstrom's macroglobulinemia, primary amyloidosis, and osteosclerotic myeloma (POEMS syndrome). Multiple myeloma is the most frequent plasma cell dyscrasia and has variable prognosis. MM has an incidence of four cases per 100,000 and accounts for 1% of all malignancies [1–3]. EMP and SP of bone are localized and typically have a better prognosis with a mean survival greater than 10 years [1]. The main prognostic indicator for these diseases is progression, as either may evolve into a disseminated MM years after the initial diagnosis. 

Multiple myeloma, also noted in the literature as “metastatic” MM, presenting as a de novo laryngeal mass is extremely rare with few reported cases [2, 4]. It is therefore crucial to distinguish an extramedullary focus of MM from a primary EMP as it affects the treatment and prognosis. EMP is defined as a localized monoclonal plasma cell tumor with absence of plasma cell infiltrate in bone marrow biopsies or blood, absence of hypercalcemia, renal failure, or anemia attributable to myeloma, no evidence of other bone lesions by imaging studies, absence or low serum or urine M protein, and normal levels of uninvolved polyclonal immunoglobulins [1]. Extraosseous tumors form a small percentage of plasma cell tumors with a greater percentage than 80 to 90% involving the head and neck [1–3]. Laryngeal involvement of extramedullary tumors is reported to be between 6% and 18% with the epiglottis, glottis, false vocal folds, aryepiglottic folds, and subglottis involved in decreasing order of frequency [2, 4]. Conversely, a diagnosis of MM requires at least 10% clonal bone marrow plasmacytosis, M protein in serum or urine (except in nonsecretory myeloma), and evidence of end-organ damage attributable to myeloma involvement (hypercalcemia, renal insufficiency, anemia, or bone disease) [1, 5]. CT with or without PET and magnetic resonance imaging can be used to further evaluate osseous and soft-tissue lesions and can further demonstrate the presence of additional, clinically occult lesions or cervical node involvement. The presence of coexisting osteolytic bone lesions in the context of previous, smoldering IgA myeloma in our patient favors the diagnosis of an extramedullary focus of multiple myeloma rather than extramedullary plasmacytoma, despite a nondiagnostic bone marrow aspirate and the absence of the detectable M protein. 

The management of plasma cell neoplasms can be performed with radiotherapy, chemotherapy, or surgery. The preferred treatment modality for SP and EMP is radiotherapy, as plasma cell neoplasms are highly radiosensitive [2]. In contrast, multiple myeloma is considered a systemic disease and is treated with chemotherapy and bone marrow transplantation [1, 2]. Our patient was treated with 30 Gy to the neck over 15 fractions with resolution of the thyroid cartilage lesion. Due to the protracted time course and mildly elevated IgA levels, chemotherapy was deferred by hematology in favor of local control with radiation. The free kappa/lambda ratio value fell within the normal range to 1.20. The patient continues to do well approximately four months following the completion of therapy.

## References

[1] McKenna RW, Kyle RA, Kuehl WM, Swerdlow S, Campo E, Lee Harris N (2008). Plasma cell neoplasms. *WHO Classification of Tumours of Haematopoietic and Lymphoid Tissue*.

[2] Nofsinger YC, Mirza N, Rowan PT, Lanza D, Weinstein G (1997). Head and neck manifestations of plasma cell neoplasms. *Laryngoscope*.

[3] Rutka J, Noyek AM, Chapnik JS, Amato D, Couter N (1985). Multiple myeloma involving the cricoid cartilage. *Journal of Otolaryngology*.

[4] Van Dyke CW, Masaryk TJ, Lavertu P (1996). Multiple myeloma involving the thyroid cartilage. *American Journal of Neuroradiology*.

[5] (2003). Criteria for the classification of monoclonal gammopathies, multiple myeloma and related disorders: a report of the International Myeloma Working Group. *British Journal of Haematology*.

